# Comparison of five different popular scoring systems to predict nonsentinel lymph node status in patients with metastatic sentinel lymph nodes: a tertiary care center experience

**DOI:** 10.1186/s40064-015-1442-4

**Published:** 2015-10-29

**Authors:** Ramazan Yıldız, Murat Urkan, Oğuz Hancerliogulları, Zafer Kılbaş, Erkan Ozturk, Mustafa Oner Mentes, Semih Gorgulu

**Affiliations:** Department of Surgery, Gulhane Military Medical Academy, Etlik, 06018 Ankara, Turkey

**Keywords:** Breast cancer, Nonsentinel lymph node status, Nomogram, Scoring system

## Abstract

Sentinel lymph node biopsy (SLNB) is the current standard of care for breast cancers with no clinically palpable axillary lymph nodes. Almost 50 % of sentinel lymph node positive patients have negative non-sentinel nodes and undergo non-therapeutic axillary dissection. Five different scoring systems, reported in the literature, were compared for their predictive ability of non-SLN involvement in patients with SLN positive breast cancer. 242 patients who underwent breast surgery and SLNB were included in the study. Of these, 70 who were confirmed to have SLN metastasis and received complementary ALND and constituted the final study population. The nomograms (MSKCC, M.D. Anderson Cancer Center, Tenon model, Stanford and Turkish) were statistically compared for their prediction of non-SLN metastasis (95 % confidence interval). We have determined only two clinicopathologic (multifocality and size of the primary tumor) situations which have a statistically significant association between SLN metastasis with using a multivariate logistic regression analysis. Multifocality (P = 0.001) and size of the primary tumor (P = 0.001) were associated with a higher probability of-SLN metastasis. No predictive model was constructed that showed good area under the curve (AUC) discrimination in the validation series. Currently published predictive models lack accuracy when applied to a different population. Multi-institutional heterogenic population studies are important to determine the exact combination of scoring systems and/or nomograms.

## Background

Axillary lymph node metastasis is the most important prognostic factor for local recurrence and survival in patients with breast cancer and is instrumental for staging and adjuvant treatment decision making (Carter et al. 1989; Rosen et al. 1989; Fisher et al. 1984).

Axillary lymph node dissection (ALND) was an important part of breast cancer treatment until the end of the 20th century (Shah-Khan and Boughey 2012). The treatment approach progressed and instead of conventional axillary dissection (level 1–3), axillary sampling (level 1–2) and latterly sentinel lymph node biopsy (SLNB) was introduced. Today, SLNB is the standard surgical approach in patients without clinical and pathologic axillary metastases. ALND, however, remains the standard method used for prognostic and therapeutic purposes in patients with sentinel lymph node (SLN) positive (Mansel et al. 2006; Goyal and Mansel 2008; Veronesi et al. 2010; Zavagno et al. 2008) breast cancer. SLNB provides sufficient information for decision making on the necessity of adjuvant therapy and prevents unnecessary axillary dissection and its related complications. On the other hand, studies investigating complementary ALND after SLNB have shown that nearly 60 % of SLN-positive patients have no metastasis in their non-sentinel lymph nodes (Turner et al. 2000; Hwang et al. 2003; Kim et al. 2006; Nos et al. 2003). For these patients, ALND has no additional therapeutic benefit. Furthermore, it causes prolonged hospitalization and increases morbidity and treatment costs (Hwang et al. 2003). Today, axillary lymph node metastases do not cause problems with systemic treatment decisions. Current guidelines recommend adjuvant therapy for patients with a primary tumor larger than 1 cm regardless of the axillary metastases (Hwang et al. 2003). Therefore, a question of whether SLN positive patients can also be followed but avoid ALND has been raised. To answer this question, the parameters and factors that would help to predict the risk of non-SLN metastasis in a given subset of patients needs to be understood (Degnim et al. 2003).

Studies with SLN positive patients focusing on the requirements of ALND are available. Giuliano AE et al’s (American College of Surgeons Oncology Group (ACOSOG) Z0011) trials have brought a new perspective on the management of axilla in breast cancer (Giuliano et al. 2010, 2011; Giuliano et al. 2012). In this study, breast cancer patients with clinically T1/T2 N0 disease who were determined to have 1–2 metastatic SLNs at or after surgery were randomized into two groups to receive complementary ALND or no additional surgical procedures. All patients underwent breast-conserving surgery, tangential breast irradiation (irradiation without axillary) and adjuvant chemotherapy was administered. There was no difference between the two groups in terms of survival. After a mean 6-year follow up, the group treated with only SLNB was found to have a 0.9 % rate of axillary recurrence. However, most of the patients in this study were postmenopausal and estrogen receptor (ER) positive low-risk patients (Pilewskie and Morrow 2014).

AMAROS (After Mapping of the Axilla: Radiotheraphy or Surgery) is another study investigating the same topic (Donker et al. 2014). Patients with primary tumor 0.5–3 cm in size and with clinically negative nodal disease were included. Patients with a positive SLN were randomized into two groups: group 1 received complementary ALND and group 2 was treated with axillary radiotherapy (RT). The axillary recurrence rate was 0.54 % in the group treated with complementary ALND and 1.03 % in the group with RT applied at 6.1 year follow-up.

There is a lot known about the risk of non-SLN metastases with the histologic features of the primary tumor. These features can be listed as histologic type, tumor grade, invasive tumor size, lymphovascular invasion, ER status, method for detection of lymph node metastasis, metastasis size, spread of extra capsular lymph node, the number of positive and negative SLN (Kim et al. 2006; Nos et al. 2003; Nadeem et al. 2014; Kamath et al. 2001). Nomograms and mathematical models using these features have been developed to estimate the risk of non-SLN metastases in patients with SLN-positive disease. The Memorial Sloan Kettering Cancer Center (MSKCC) nomogram was published in 2003 (Van Zee et al. 2003) and subsequently, different studies have investigated the validation of this nomogram. Some of these studies reported controversial results (Unal et al. 2008) and limitations (Nadeem et al. 2014) and consequently, several additional nomograms and scoring systems were introduced relying on mathematical calculations using a number of variables [University of Texas MD Anderson Cancer Center (MDACC) (Mittendorf et al. 2012) and include Tenon (Barranger et al. 2005), Stanford (Kohrt et al. 2008) and Turkish (Gur et al. 2010) systems (Table 1)].

**Table 1:** Variables used in all five nomograms

Variables	Nomograms				
	MSKCC	MDACC	Tenon	Stanford	Turkish
Frozen section	+				
Pathologic size of tumor	+	+	+	+	
Grade of tumor	+				
Number of positive SLNs	+	+			
Number of negative SLNs	+				
Number of SLNs(total)		+			
Type of tumor	+	+			
Detection method of SLN	+				
Lymphovascular invasion	+	+		+	+
Multifocality	+				
ER –Positive	+				
Micro-macrometastasis			+	+	
Overall metastasis size in SLNS		+			+
Proportion of involved SLNs/all SLNs			+		+
Extranodal extension		+			

*MSKCC* Memorial Sloan Kettering Cancer Center, *MDACC* University of Texas MD Anderson Cancer Center, *SLN* sentinel lymph node

The aim of this study is to investigate the accuracy of these five different nomograms and scoring systems (MSKCC, MDACC, Tenon, Stanford, Turkish) in our patient population.

## Methods

The study design was approved by the Ethics Committee of Gulhane Military Medical Academy (02/42, Sept 2014). In total, medical records of 685 patients who had surgery for invasive breast cancer between years 2004–2012 at a tertiary care center were examined retrospectively. Some 242 patients with clinical T1 to T3, N0 disease who underwent breast-conserving surgery or mastectomy and SLNB (with no history of axillary surgery or neoadjuvant therapy) were included in the study. Of these, 70 were detected to have SLN metastasis, received complementary ALND and constituted the final investigated study population. Patients’ age, tumor size, tumor type, nuclear grade, tumor location, multifocality, estrogen receptor, progesterone and C-erb B2 receptor, lymphovascular and perineural invasion, SLNB method, number of exemplified SLNs, number of positive SLNs, size of metastasis in SLN, micro metastasis, the presence of extra capsular extension, the number of lymph nodes harvested during complementary ALND and non-SLN metastasis were recorded (Table 2). Then, the collected data were used to calculate the risk of non-SLN metastasis with the nomograms included in this study.

**Table 2:** Descriptive characteristics of the study group (n = 70)

Characteristics of patients	n (%)
Age (years)	
≤50	36 (51.4 %)
>50	34 (48.6 %)
Pathologic tumor size (cm)	
≤2	28 (40 %)
2–5	40 (57.1 %)
>5	2 (2.9 %)
Tumor type	
Invasive ductal carcinoma	60 (85.7 %)
Invasive lobular carcinoma	6 (8.6 %)
Other	4 (5.7 %)
Nuclear grade	
1	0
2	34 (48.6 %)
3	36 (51.4 %)
Localization of tumor	
Upper outer quadrant	22 (31.4 %)
Upper inner quadrant	8 (11.4 %)
Lower outer quadrant	6 (8.6 %)
Lower İnner quadrant	4 (5.7 %)
Central	5 (7.2 %)
Unknown	25 (35.7 %)
Multifocality	
Yes	14 (20 %)
No	56 (80 %)
Estrogen receptor	
Positive	54 (77.1 %)
Negative	16 (22.9 %)
Progesterone receptor	
Positive	58 (82.9 %)
Negative	12 (17.1 %)
c-erb B2 receptor	
Positive	17 (24.3 %)
Negative	53 (75.7 %)
Lymphovascular invasion	
Yes	11 (15.7 %)
No	59 (84.3 %)
Perineural invasion	
Yes	2 (2.8 %)
No	68 (97.2 %)
Method of SLNB	
Isosulfan blue	53 (75.7 %)
Tc99 m sulfur colloid	10 (14.3 %)
Combined	7 (10 %)
Number of SLNs	
1	24 (34.2 %)
2	16 (22.8 %)
3	13 (18.6 %)
>3	17 (24.3 %)
Number of positive SLN	
1	39 (55.7 %)
2	20 (28.6 %)
3	9 (12.8 %)
>3	2 (2.9 %)
Proportion of involved SLNs among all SLNs, number of SLNs	
1	40 (57.1 %)
0.75	5 (7.1 %)
0.66	4 (5.7 %)
0.50	8 (11.4 %)
0.42	1 (1.4 %)
0.40	1 (1.4 %)
0.33	5 (7.1 %)
0.28	1 (1.4 %)
0.25	5 (7.1 %)
Micrometastases in SLNs	
Yes	13 (18.6 %)
No	57 (81.4 %)
Extracapsular extansion	
Yes	6 (8.6 %)
No	64 (91.4 %)
Additional non-SLN metastases	
Present	34 (48.6 %)
Absent	36 (51.4 %)
Number of non-SLNs with additional metastases	
1	30/34 (88.2 %)
2	2/34 (5.9 %)
3	1/34 (2.9 %)
>3	1/34 (2.9 %)

*SLNB* sentinel lymph node biopsy, *SLN* sentinel lymph node

## SLNB method and pathologic evaluation

SLNB was performed after injection of 4 mL of 1 % blue dye isosulfur or Tc99 m sulfur colloid. All of the extracted sentinel lymph nodes were sent for frozen section examination during surgery. During frozen section examination, 1 cm or smaller lymph nodes were bisected to the long axis and so both were prepared for imprint cytology. Later, one of the pieces from the frozen sections was reserved for routine examination. The frozen section and imprint preparations were examined under a microscope through hematoxylin and eosin (H & E) staining. Lymph nodes larger than 1 cm were cut into 3 mm slices and examined in the same way. Then, imprint preparations from all sides were examined with a light microscope. Frozen sections from imprint preparations suspected of metastasis were examined again. All excised lymph nodes were evaluated by H & E staining and immunohistochemical analysis was performed when necessary.

## Nomograms

To predict the likelihood of non-SLN metastases, the five different nomograms were sequentially applied. For MSKCC, Stanford University and MDACC nomograms, calculators available on the following web sites were used, respectively:

- (http://nomograms.mskcc.org/Breast/BreastAdditionalNonSLNMetastasesPage.aspx),
- (http://www3-hrpdcc.stanford.edu/nsln-calculator/),
- (http://www3.mdanderson.org/app/medcalc/bc_nomogram2/index.cfm?pagename=nsln).

For Tenon and Turkısh nomograms, the formula described in the relevant publications were utilized (Barranger et al. 2005); (Gur et al. 2010).

The predicted change of non-SLN metastases was compared with the actual presence/absence of metastatic non-SLNs after complementary ALND. ROC curves of these nomograms were created and the AUC was calculated with the aim of predicting the presence of axillary lymph node metastasis. The AUC results were grouped under the classification of a 0.5 value as insufficient, 0.7–0.8 as good and 0.8–0.9 as very good. Then, the nomograms were compared in terms of the statistical prediction of non-SLN metastasis (95 % confidence interval).

### Statistical analysis

The statistical analyses were performed using the SPSS statistical software (version 15.0, *IBM, USA*). Results are given as mean standard deviation. Differences between the study groups were analyzed and Chi square test was used to compare the variables. Statistical significance was set at 0.05. The areas under (AUC) the receiver operating characteristic curve (ROC) were used to describe the performance of the diagnostic value of each nomogram. A model with a ROC of 0.5 did not turn out to be sufficient. A model with a ROC of 0.7–0.8 was considered good and a ROC of 0.81–0.9 was excellent. The accuracy, sensitivity, specificity, positive and negative predictive values of each nomogram was calculated through analysis methods.

## Results

### Descriptive characteristics

Twenty-nine percent of patients (n = 70) were determined to have a SLNs positive. The mean patient age was 51 ± 14 years. The mean SLN number was 3 ± 1 and the mean overall metastasis size was 11 ± 7. The metastasis of SLN was confirmed as micrometastasis in 13 (18.6 %) patients and macrometastasis in 57 (81.4 %) patients. The mean tumor size was 25 ± 12 mm. Multifocality was present in 20 % (n = 14) and lymphovascular invasion was present in 15.7 % (n = 11) of patients. Extra nodal invasion was present in 8.6 % (n = 6) of patients. In our SLNs positive data, 34 (48.6 %) patients had positive axillary non-SLN metastases. The mean number of involved non-SLN metastases was 2 ± 1. Other clinicopathologic characteristics of this study are given in Table 2.

Statistical analysis showed a significant association between SLN metastasis and variables preoperative ultrasonographic findings of the pathologic size of the primary tumor (P < 0.001), type of tumor (P = 0.029), multifocality (P = 0.005), presence of LVI (P = 0.007), and tumor grade (P = 0.003). There were no other clinicopathologic significant statistical associations with the likelihood of SLN metastasis. However, we have determined just two clinicopathologic (multifocality and size of the primary tumor) situations which have a statistically significant association between SLN metastasis with using a multivariate logistic regression analysis. Multifocality (P = 0.001) and size of the primary tumor (P = 0.001) were associated with a higher probability of-SLN metastasis. Statistical analysis was performed with variables for the determination of the Non-SLN positivity. However, there was no statistically significant association between non-SLN positivity and the other variables.

### AUC values of the nomograms

**Fig. 1:**
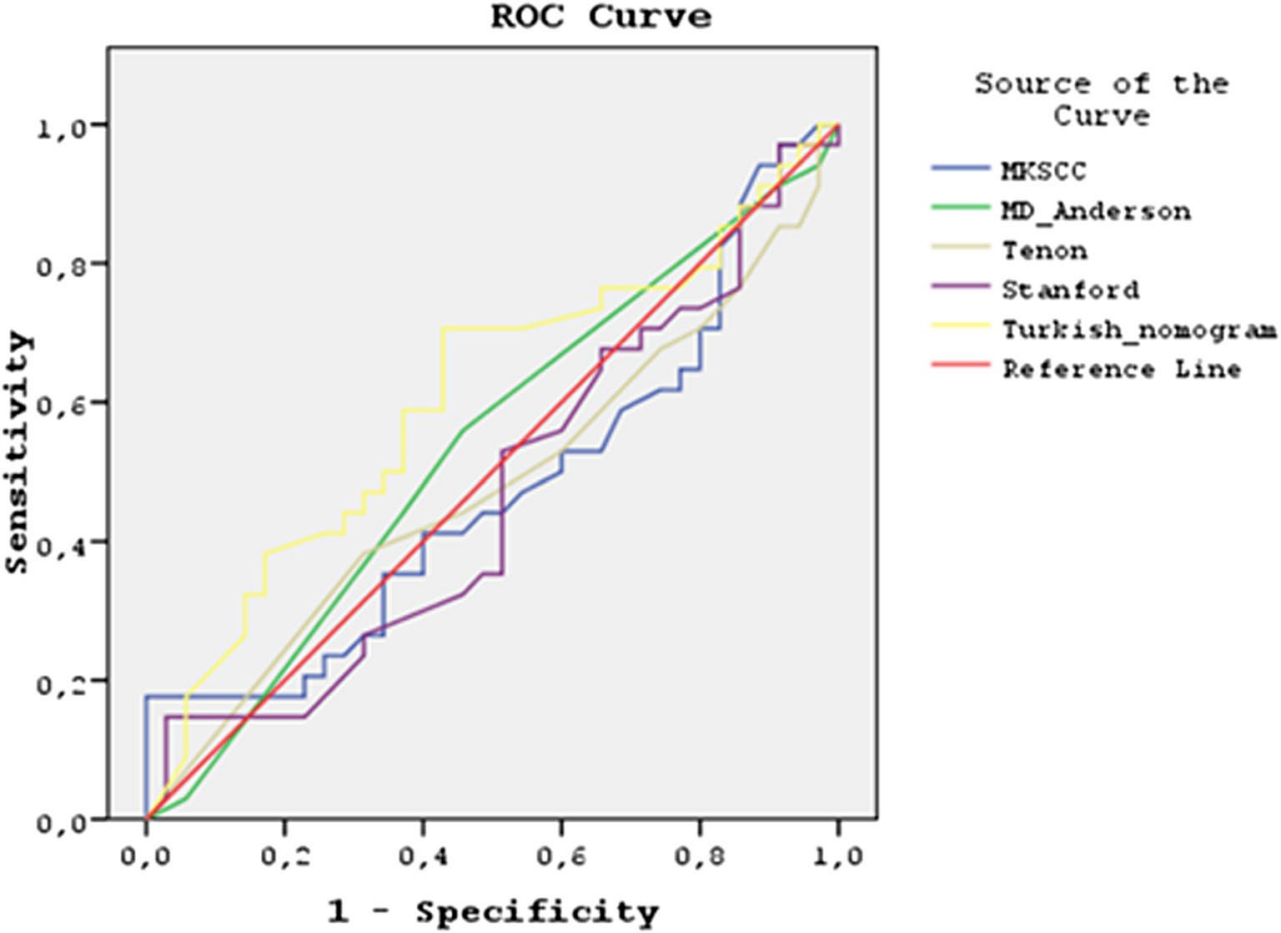
AUC values for different nomograms. *AUC* area under the curve, *MSKCC* Memorial Sloan Kettering Cancer Center, *ROC* receiver operating characteristic

We determined five scoring systems (MSKCC, M.D. Anderson Cancer Center Tenon model, Stanford and Turkish) which were adopted for the determination of the Non-SLN metastasis. The ROC curve of these scoring systems and nomograms (MSKCC, MD Anderson, Tenon, Stanford and Turkish) were drawn using substantiation (n: 70) datasets. Then, discrimination of score systems and nomograms was assessed by generating Receiver Operating Characteristic (ROC) curves, calculating the area under the receiver operating characteristic curve (AUC) with a 95 % confidence interval (95 % CI) for each model. The AUC varies between 0.5 and 1.0, and a value greater than 0.70 was considered to demonstrate a good discrimination. The AUCs in the validation datasets were 0.525, 0.534, 0.520, 0.534 and 0.6050 (MSKCC, M.D. Anderson Cancer Center Tenon model, Stanford and Turkish) respectively (Fig. 1). When an identical validation dataset was applied, the AUCs of the five models were not significantly different. The AUCs of Turkısh scoring system was slightly higher than the other four scoring systems. However, no statistically significant difference was observed between Turkısh and the other scoring systems (Table 3). The values for predicting the probability of having further metastases in NSLNs for the MSKCC, MDACC, Tenon, Stanford and Turkish models were 45, 54.3, 45, 48.6, and 61.5 %, respectively. And, for negative predictive probability was 46.7, 57.11, 46.7, 51.5, and 67.7 % respectively. Positive and negative predicted probability (P) values (%) and their sensitivity and specificity values are shown in Table 3.

**Table 3:** Five different nomograms/models for predicting nonsentinel node status after tumor-positive SNB

	AUC	PPV (%)	NPV (%)	Sensitivity (%)	Specificity (%)	P
MSKCC	0.525	45	46.7	52.9	38.9	0.723
Md Anderson	0.534	54.3	57.11	55.9	55.5	0.623
Tenon	0.520	45	46.7	52.9	38.9	0.778
Stanford	0.534	48.6	51.5	52.9	47.2	0.627
Turkish nomogram	0.605	61.5	67.7	29.41	41.66	0.135

*PPV* positive predictive value, *NPV* negative predictive value

## Discussion

Determination of axillary nodal status is still the most important prognostic factor to accurately stage and locally control breast cancer and to reduce the change of locoregional recurrence. ALND had been the standard approach for patients with invasive breast cancer up until the 2000s. However, this approach causes severe morbidities (acute and late) with complications such as limited shoulder movement (1 %), pain, lymphedema (2–38 %), and loss of sensations etc. (Mansel et al. 2006). ALND also helps determine those who would benefit from adjuvant systematic treatment (Beriwal et al. 2008). On the other hand, there are some controversial opinions related to ANLD. For example, a trial (Z0011) by American College of Surgeons Oncology Group revealed that omitting ALND did not cause an increase in the rate of local (p = 0.11), or regional (p = 0.45) recurrence and did not result in inferior survival (Giuliano et al. 2010). Therefore, during the last decade SLNB has been used to determine the axillary lymph node status in patient with invasive breast cancer (Veronesi et al. 2003).

Sentinel lymph node biopsy (SLNB), is now widely adopted as the standard of care for the definition of axillary node staging. When SLNB shows no metastasis, the patient’s axilla does not require any further management other than surveillance and the treatment decision is made according to other parameters. In other words, if the SLNB is positive, this prompts a big question for clinicians. Some studies have revealed that nearly 50–60 % of patients with tumor-positive SLNs have no further metastases in the ALND (Reynolds et al. 1999; Kim et al. 2006). Moreover, the beneficial effect of ALND and its value in the facilitation of therapy strategy has been a controversial topic. If the SLNs harbor only micrometastasis or isolated tumor cells, the risk for additional metastases in the non-sentinel LNs decreases to 10–15 %. Due to all the factors mentioned above, the requirement for ALND in all patients with positive SLNs has been questioned. Moreover, multiple reasons such as receiving adjuvant systemic therapy and/or whole-breast irradiation have been suggested if ALND is not needed in node-positive breast (Greenberg et al. 2008). So, this led us to identify the subset of patients with positive SLNs who would be at low risk of developing NSLN metastases.

Several scoring systems and mathematical models have been described to prognosticate the probability of NSLN metastases. Some of these nomograms seem to provide a useful predictive tool for NSLN metastases. While they are known to be effective in predicting the risk of NSLN disease in homogeneous populations in which they were created, tested and validated, the results from validation studies implemented on heterogeneous groups or in other populations have not demonstrated similar success. For example, MSKCC (from Breast Service of Memorial Sloan-Kettering Cancer Center) seems to be a useful predictive tool in the North America (Smidt et al. 2005), while when applied to other study populations it was less satisfactory. It requires ten variables (frozen section, tumor size, tumor type, tumor grade, number of positive SLNs, SLN method of detection, number of negative SLNs, lymphovascular invasion status, multifocality, and estrogen receptor status) to determine a patient’s risk of having NSLN metastasis. *Klar* et al. used the MSKCC nomogram in 98 German breast cancer patients and found only a 0.58 AUC value. The study by Dauphine on 51 patients reported an AUC of 0.63 and concluded that the MSKCC nomogram could not accurately predict the probability of non-SLN metastasis at their institution and in their populations (Klar et al. 2008; Dauphine et al. 2007). Our results showed a moderate power of discrimination with the AUC of 0.525 for the MSKCC nomogram, which had some limitations as a suitable discriminator of NSLNM in SLN positive patients. Especially, clinicians must be careful when using the MSKCC in patients with micrometastasis because of the fact that it does not include the predictive parameter of the size of SLN metastasis (Alran et al. 2007). Again, in our study, thirteen (18.6 %) patients had micrometastasis in their SLNs and 57 (81.4 %) had macrometastasis. Consequently, MSKCC could not be validated as a predictive nomogram in the micrometastasis group.

*Barranger* et al. (Barranger et al. 2005) described a scoring system (in the Tenon Hospital) after 2 years (2005) MSKCC which had previously been described as a first nomogram in 2003 by Van Zee et al. (Coutant et al. 2009). This scoring system consisted of three variables; the ratio of positive SLNs to the total number of removed SLNs; the size of metastasis (micro- or macrometastasis) in the SLN; and the histopathological tumor size. The strongest predicting factor for NSLN metastases was the size of the SLNs metastases, while the size of the SLN metastasis was not evaluated by MSKCC. Positive SLNB patients have been scored with the range of 0–7. A patient’s score between ≤3.5 and ≤4, had an approximately 95 % chance (respectively) of tumor-negative NSLN metastases. The Tenon model nomogram was validated by *Coutant et al*’s prospective multicenter study (Coutant et al. 2009) and demonstrated that the Tenon score was accurate with 92.1 % sensitivity, 70.1 % specificity, 95.8 % negative predictive value, 54.7 % positive predictive value, and 0.82 AUC, for predicting non-SN status in patient with SLNB positive breast cancer patients. In our study population, the Tenon method was not a good predictor for non-SN status with a low sensitivity and specificity (29.41, 41.66 %) respectively. Also, it’s negative predictive value (46.7 %), positive predictive value (45 %) and AUC (0.520) were under the borderline. This was not a good discriminator for our breast cancer populations and was unable to predict of the probability of NSLNM.

Another validated scoring system included in our study is the MDACC (MD Anderson Cancer Centre) nomogram. It was developed by M.D Anderson Cancer Center in 2009 to predict the likelihood of non-SLN metastasis at retrospective evaluation of clinicopathological data from 131 patients with a positive SLN biopsy (Hwang et al. 2003). It was designed to incorporate four parameters (tumor size >2 mm = 1, number of SLNs examined ≥3 = −2, largest SLN metastases >2 mm = 2, presence of LVI = 1) that independently predict non-SLN metastases. All parameters were added to achieve a final score for each patient. The final score ranged from (−2) to (4). *Hwang RF* et al. revealed that primary tumor >2 cm (P = .009), SLN metastasis >2 mm (P = .024), and lymphovascular invasion (P = .028) were independent predictors of positive NSLNs. However, our population’s multivariate analysis was not given the same statistical significant consequence as NSLNs metastasis prediction. But, we found statistically significant results with tumor size (P < 0.001), multifocality (P = 0.005) and LVI (P = 0.007) which are related to the prediction of Non-SLNs metastasis.

Suggestion on another novel scoring system (Stanford Online Calculator) was reported in 2008, in conjunction with an online calculation method which uses multivariate logistic regression analysis powered by the Classification and Regression Trees using data from the Bay Area SLN Database. It evolved from retrospective information of 285 patients with positive SLNs who underwent ALND. The Stanford scoring system uses three variables: size of SLN metastasis, primary breast tumor size and the presence or absence of LVI. *Scow JS et al.* externally validated this scoring system, and obtained 0.72 AUC (95 % CI 0.67–0.77) for their patient population (Scow et al. 2009). They also suggested that this system showed good performance in their patient population, but this was not superior to the AUCs of the MSKCC (0.74). The AUCs of the two models were not significantly different in our study (P = 0.13) and there was no significant differences between these two system’s AUCs (0.525, 0.534) respectively. Moreover, unlike *Scow JS et. al*’s study, we did not achieve a good performance for the validation of Stanford model and MSKCC.

We analyzed the Turkish monogram which was developed in a patient population that was more likely to have similar clinical and patient characteristics to our group of subjects. *Gur A.S* et al. (2010) described the Turkish nomogram as a result of Turkish Federation of Breast Disease Associations Protocol MF08-01 investigation (Gur et al. 2010). They identified three predictors by multivariate analysis: SLN metastasis size, proportion of involved SLNs among all SLNs and lymphovascular invasion (LVI). They validated MSKCC, Cambridge, Stanford and the Tenon models with their breast cancer population. The AUC values were 0.705, 0.711, 0.730, and 0.582 respectively. They suggested that; the MSKCC, Cambridge, and Stanford nomograms were good discriminators of NSLNM in SLN positive breast cancer patients. However, in their study the Turkish nomogram provided the best result with the 0.8023 AUC value. The Turkish model was further validated with two different populations by *Hidar S* et al. and *Nadeem RM* et al. with good predictive value of AUC (0.75, 0.70) respectively (Nadeem et al. 2014; Hidar et al. 2011). Our study did not detect the same AUC value (0.605) as the Turkish model however this result is the highest AUC value among all the nomograms applied to our population.

The limitation of our study is that it is a retrospective study using a single and small population. Despite these limitations, our scoring system provides a good perspective about the prediction of non-SLN status.

In summary, the validation of previously published predictive models showed that these nomograms performed well at the center where they were developed, but it seemed to lose accuracy when applied to a different population. Of course, scoring systems supply beneficial information regarding the likelihood of metastasis in nonsentinel nodes, but predictability remains a big problem. The use of scoring systems or nomograms that are developed at other institutions should be applied with caution when evaluating the patients with a positive SLNB. Multi-institutional with heterogenic population studies should be developed to determine the exact combination of scoring systems and/or nomograms to provide the most accurate prognostic value.
